# Whole-genome sequencing and integrative genomic data-driven profiling of Carbapenems-resistant *Klebsiella pneumoniae* isolated from human urine samples in the Iraq

**DOI:** 10.1016/j.dib.2026.112712

**Published:** 2026-03-24

**Authors:** Hijran Mohsen Meshjel, Safaa Al-Deen Ahamed Shanter Al-Qaysi

**Affiliations:** Department of Biology, College of Science for Women, University of Baghdad, Baghdad, Iraq

**Keywords:** Klebsiella pneumoniae, Carbapenem resistance, Whole-genome sequencing, Multidrug resistance, Virulence factors

## Abstract

Here, we present whole genome sequencing (WGS) data of clinical *Klebsiella pneumoniae* and *K. quasipneumoniae* isolates obtained from urine samples collected between October 2024 and January 2025 from patients in hospitals in Baghdad, Iraq. A total of 30 *K. pneumoniae* isolates were initially recovered and screened using antimicrobial susceptibility testing, from which one carbapenem-resistance isolate (isolate 5) and one carbapenem- susceptible isolate (isolate 6) were selected for genome sequencing. Genomic DNA was extracted, sequenced using the illumine MiSeq platform, and assembled de novo. The resulting genome assembled were annotated using NCBI prokaryotic genome annotation pipeline and further analysed using the PATRIC platform. The dataset includes annotated genome assemblies with identified carbapenemase genes (including *blaNDM*-5, *blaOXA*-48, and *blaOXA*-181), additional antimicrobial resistance determinants, predicted virulence-associated genes, mobile genetic elements, and multilocus sequence typing results. Isolate 5 was assigned to sequence type ST14 (*K. pneumoniae*), while isolate 6 was assigned to ST7604 (*K. quasipneumoniae*). These genome data provide a curated genomic resource that can be reused for comparative genomics, antimicrobial resistance surveillance, and phylogenomic analysis of *Klebsiella* species from clinical setting in Iraq.

Specifications Table

**Table:** 

Subject	Biology
Specific subject area	Clinical Genetics, Microbial genomics, Bacteriology
Type of data	Whole genome sequencing data, assembled and annotated draft genome of the isolates *Klebsiella pneumoniae* ST14 and *Klebsiella quasipneumoniae* ST7604
Data collection	Clinical *K. penumoniae* isolates were collected from urine samples of patients with urinary tract infections in Baghdad, Iraq. After bacterial identification and antimicrobial susceptibility testing, genomic DNA was extracted from selected isolates and subjected to whole-genome sequencing using the Illumina MiSeq Platform, Raw sequencing reads were quality-checked, *de novo* assembled, and subsequently annotated and analyzed using established bioinformatics pipelines to characterized genomic features, antimicrobial resistance determents, and virulence-associated genes.
Data source location	University of Baghdad, College of Science for Women, Department of Biology. City: Baghdad, Al-Jadriya; Country: Iraq.
Data accessibility	The Dataset are hosted in a public repository in Mendeley Data: (DOI:10.17632/nc7dsgtvkk.1) Repository name: National Centre for Biotechnology information (NCBI) Klebsiella pneumoniae ST14 isolate: Bioproject Accession Number: PRJNA1371618 Sequence Read Archive Accession Number (SRA) SRR27266164 Biosample: SAMN53505489 Klebsiella quasipneumoniae ST7604 isolate: Bioproject Accession Number: PRJNA1371618 Sequence Read Archive Accession Number (SRA) SRP650226 Biosample Accession number: SAMN53505490 Direct URL to data: https://www.ncbi.nlm.nih.gov/bioproject/?term=PRJNA1371618 https://www.ncbi.nlm.nih.gov/biosample/?term=SAMN53505490 https://www.ncbi.nlm.nih.gov/sra/?term=SRX31289847 https://www.ncbi.nlm.nih.gov/sra/?term=SRX31289846 https://www.ncbi.nlm.nih.gov/sra/?term=SAMN53505490 https://www.ncbi.nlm.nih.gov/sra/?term=SRR36255186 https://www.ncbi.nlm.nih.gov/sra/?term=SRR36255187
Related research article	None

## 1 Value of the Data

• The whole genome sequence of *K. pneumoniae* can support a comparative genome study of these bacteria isolated from patients infected with urinary tract infection in different hospitals of Baghdad city, Iraq.
• The present data of whole genome sequencing described the antibiotic resistance and predicts the antimicrobial resistance profile for further drug development and treatment of infections.
• The functional annotation of isolated *K. pneumoniae* isolates a wide plethora of genetic elements either involves AMR, Virulence, sequence typing or other primary and secondary metabolic pathway.
• The genomic data helped us to analyze phylogeny based on whole genome sequencing of CRKp and the susceptible isolates.

## 2 Background

The increasing prevalence of antibiotic-resistant clinical bacterial isolates in health systems worldwide is concerning [1,2]. Studying and understanding the genetic factors underlying antibiotic resistance is crucial for preventing the dissemination of multidrug-resistant (MDR) bacteria [3]. Among these MDR bacteria, *K. pneumoniae* is considered one of the six major causes of nosocomial infections and antimicrobial resistance [4]. As an opportunistic pathogen, *K. pneumoniae* is a Gram-negative bacillus of the Enterobacterales family and primarily affects immunocompromised individuals, particularly hospitalized patients. Many infections, including sepsis, bacteraemia, pneumonia, and urinary tract infections, are caused by *K. pneumoniae* [5]. Some *K. pneumoniae* capsular serotypes that produce large amount of capsular polysaccharide than others are classified as hypervirulent strains. These strains produce a markedly mucoid capsule that increases resistance to phagocytosis and immune clearance consequently, they can cause invasive diseases requiring hospitalization [6]. They display enhanced virulence and immune evasion. As producers of *K. pneumoniae* carbapenemase (KPC), these strains can hydrolyzed many β-lactam antibiotics, particularly carbapenem, which are increasingly used to treat hospital-acquired infections and infection unresponsive to other agent [7]. Infectious disease experts are particularly concerned about the increasing prevalence of multidrug-resistant *K. penumoniae* (MDR-KP) and carbapenem-resistant *K pneumoniae* (CR-KP) because of limited treatment options. At the molecular level, resistance to carbapenem in *K. penumoniae* is primarily mediated by carbapenemase-encoding genes, such as *blaNDM-5* and *blaOXA-48*, which confer resistance through enzymatic hydrolysis of carbapenms and β-lactam antibiotics. The co-occurrence of these genes within a single isolate is particular epidemiological relevance, as it may result in broad-spectrum β-lactam resistance and compromise the activity of multiple antimicrobial agents. From a genomic perspective, the identification of such resistance determinates provides essential data for understanding resistance mechanisms and support antimicrobial resistance surveillance and infection control strategies [8]. The dissemination of such resistance determinants is closely linked to the population structure of *K. pneumoniae* is genetically diverse yet clonal, with distinct clonal groups (CGs) that include multidrug-resistant and hypervirulent (Hv) linages [9,10].

## 3 Data Description

### 3.1 Antimicrobial susceptibility patterns

A total of 30 isolates of *K. pneumoniae* were isolated and identified from urine samples subjected to antimicrobial susceptibility testing. Among the examined isolates, 100 % (30/30) of the isolates were resistant to cefotaxime, ceftazidime, and ampicillin. Additionally, 90 % (27/30) of the isolates were resistant to ceftriaxone. While, Resistant to trimethoprim-sulfamethoxazole was determined in 86.7 % (26/30) of the isolates. Resistant to amikacin and azteronamin was detected in 83.3 % (25/30) of the isolates. Furthermore, 80 % (24/30) of the isolates were resistant to ciprofloxacin and nitrofurantoin. In addition, 70 % (21/30) were resistance to gentamicin, and 73.3 % (22/30) were resistant to impenem. The lowest resistant rate was recorded for meropenem at 66.7 % (20/30) (Table 1).

**Table 1:** Antimicrobial susceptibility patterns of *K. pneumoniae* isolates, determined based on inhibition zone diameter interpretation according to the CLSI, 2023 guidelines.

Antibiotic	class of antibiotic	Susceptible, n (%)	Intermediate, n (%)	Resistant, n (%)
IMI	Carbapenem (β-lactam)	1(3.7)	7(23.3)	22(73.3)
MEM	Carbapenem (β-lactam)	8(29.6)	2(6.7)	20(66.7)
AMP	Penicillin (β-Lactam)	0(0.0)	0(0.0)	30(100)
ATM	Monobactm (β-Lactam)	2(6.7)	4(13.3)	25(83.3)
CRO	Cephalosporin	1(3.7)	2(6.7)	27(90.0)
CTX	Cephalosporin	0(0.0)	0(0.0)	30(100)
AK	Aminoglycoside	3(10.0)	2(11.1)	25(83.3)
CN	Aminoglycoside	(0.0)	9(30.0)	21(70.0)
LEV	Fluoroquinolone	3(10.0)	6(20.0)	21(70.0)
CIP	Fluoroquinolone	1(3.7)	5(16.7)	24(80.0)
SXT	Folate pathway inhibitor	2(6.7)	4(13.3)	26(86,7)
CAZ	Cephalosporin	0(0.0)	0(0.0)	30(100)
F	Nitrofuran	3(10.0)	3(10.0)	24(80.0)

AK = amikacin; AMP = ampicillin; ATM = aztreonam; CAZ = ceftazidime; CIP = ciprofloxacin; CRO = ceftriaxone; CTX = cefotaxime; CN = gentamicin; *F* = nitrofurantoin; IMI = imipenem; LEV = levofloxacin; MEM = meropenem; SXT = trimethoprim/sulphamethoxazole.

### 3.2 Whole-genome sequencing features

Following *de novo* assembly, the genome sizes of isolates 5 and 6 *K. pneumoniae* were 5739,447 bp with G+C contents of 57.1 % and 5451,888 bp at 58.0 % respectively, Assembly gaps were closed through PCR amplification followed by Sanger sequencing. The assembled genomes consisted of contig numbers of 155 contigs for isolate 5 and 44 contigs for isolate 6, respectively, with N50 values of 160,000 bp and 720,098 bp, respectively (Table 2). Genome annotation was performed using the NCBI prokaryotic Genome Automatic Annotation Pipeline (PGAAP), which predicted 5681 and 5320 protein-coding sequences, 77 and 78 tRNA genes, and rRNA genes for isolates 5 and 6 respectively. To determine the genomic population structure, multilocus sequence typing (MLST) was performed based on the seven housekeeping genes *(gapA, infB, mdh, pgi, phoE, rpoB and tonB).* The analysis assigned isolate 5 to sequence type ST4, whereas isolate 6 was classified as sequence type ST7604. MLST results are summarized in Table 3.

**Table 2:** Multi locus sequencing type of local *K. pneumoniae* isolates.

*Klebsiella isolates*	Sequence Type	Locus	Locus	Locus	Locus	Locus	Locus	Locus
		1	2	3	4	5	6	7
*K. penumoniae* isolate 5	ST14	gapA_1	infB_6	mdh_1	pgi_1	phoE_1	rpoB_1	tonB_1
*K. quasipenumoniae* isolate 6	ST7604	gapA_17*	infB_19	mdh_79	pgi_20	phoE_108	rpoB_18	tonB_142

Notes: * alleles with <100 % identity found; * gapA: Novel allele, ST may indicate nearest ST.

**Table 3:** Genomic features of the two *K. pneumoniae* isolates (ST14 and ST7604) isolated from urine specimens*.*

Characteristic	*K. pneumoniae* 5 (ST14)	*K. quasipneumoniae* 6 (ST7604)
**Genome ID**	570.7429	570.7430
**Genome length (bp)**	5739,447	5451,888
**G+C content (%)**	57.12	58.00
**Number of contigs**	155	44
**Contig L50**	11	5
**Contig N50 (bp)**	160,000	720,098
**Genome assembly quality**	Good	Good
**Predicted CDSs**	5681	5320
**tRNA genes**	77	78
**rRNA operons**	3	4
**Transporter proteins**	591	566
**Predicted drug targets**	318	310
**Completeness (%)**	100	100
**Contamination (%)**	0.0	0.3

### 3.3 Comparative whole-genome visualization of two local *K. pneumoniae* isolates

Circular genome maps (Circos plots) were generated for the sequenced isolates No 5 and 6 to provide an overview of their genomic architecture and functional annotations. Both genomes display a circular chromosome structure of *K. pneumoniae*, with coding sequences distributed across the outermost rings and distribution along the genome. The inner tracks represent GC content and GC skew distribution across the chromosome. Radial tracks indicate the genomic locations of antimicrobial resistance (AMR) genes, virulence-associated genes, and mobile genetic elements identification during genome annotation.

**Fig. 1:**
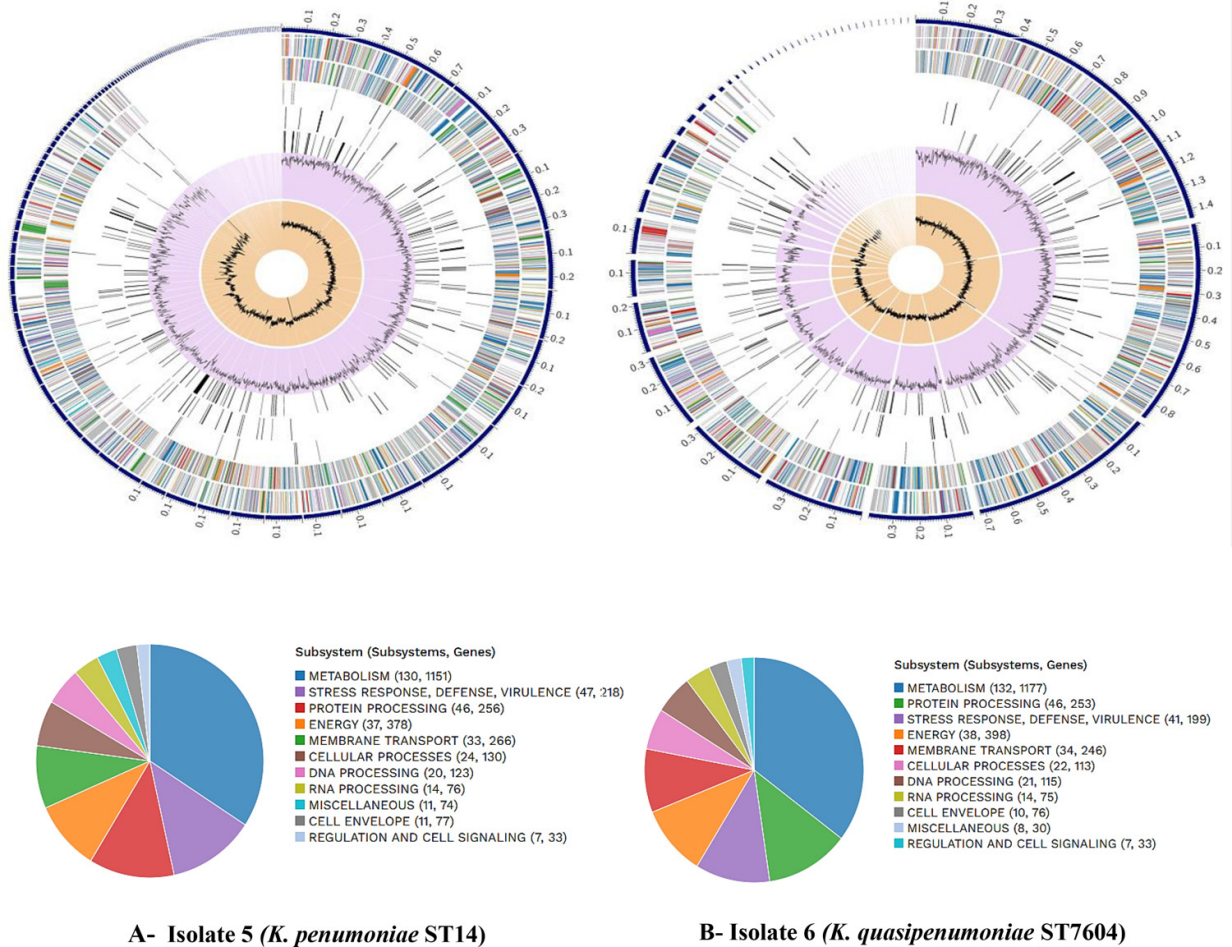
Circular genome map (Circos plot) of local isolates (**A**) isolate 5 (*K. penumoniae* ST14) (**B**) Isolate 6 (*K. quasipenumoniae* ST7604). From the outer to inner: coding sequences (CDSs) display on forward and reverse strand, genome coordinates, antimicrobial resistance genes, virulence-associated genes, and mobile genetic elements. GC content (purple) and GC skew (orange) are shown in the innermost rings. Black radial markers indicate the positions of resistance and virulence genes. Functional subsystems are displayed using color-coded annotation.

The functional categorization of annotated genes in the examined isolates of *K. pneumoniae* ST14 and *K. quasipneumoniae* ST7604 includes subsystem related to core metabolism including key biosynthetic pathways, the tricarboxylic acid (TCA) cycle, and glycolytic pathway, as well as membrane transport and translational modification. Additional function categories include protein processing functions such as folding, post-translation modification, and degradation, together with genes associated with cell envelope components. The data set also includes annotation related to stress response, RNA and DNA processing, cellular processes, regulatory and signaling function, as well as genes not assigned to a defined subsystem. All these genomic features are illustrated in Fig.1.

### 3.4 Prevalence of antimicrobial resistance genes

Whole-genome sequencing (WGS) analysis identified multiple acquired antimicrobial resistance (AMR) genes in the isolates 5 (*K. pneumoniae* ST14) and isolate 6 (*K. quasipneumoniae* ST7604) with a total of 74 and 64 acquired AMR-associated genes detected, respectively. The identified genes are associated with resistance to several classes of antibiotics, including β-lactams, aminoglycosides, fluoroquinolones, tetracycline, sulfonamides, and several efflux system. several resistance determinates were detected in both tested isolates, including (e.g*. acc(3*)*-lld, sul1, rmtB*, and multidrug efflux pumps such as *AcrAB-TolC, AcrAD-TolC, AcrEF-TolC, EmrAB-TolC, MacAB, MdtABC/TolC*. In addatioin, some genes were detected exclusively in one isolates, including *cmlA* and *qacE* in isolate No 5 and *tet(D)* in isolate No 6, The distribution of AMR genes identified in the tow isolates is summarized in Table 4. Carbapenemase-associated genes, including (*blaOXA-181, blaNDM*-5), together with porine-related gene (OmpK35), were detected in isolates 5 (K. pneumoniae ST14). In contrast, isolate 6 (*K. quasipneumoniae* ST7604) contained β-lactamase genes including, (*OKP-A-17, SHV-12*) as well as plasmid replicons sequences (lncFB (K)/lncFII (K) and lncY), as shown in Table 4.

**Table 4:** Predicted antimicrobial resistance genes identified in isolate 5 (*K. pneumoniae* ST14) and isolate 6 (*K. quasipneumoniae* ST7604).

Antimicrobial resistance mechanism	Isolate 5 (*K. pneumoniae* ST14)	Isolate 6 (*K. quasipneumoniae* ST7604)
Enzyme responsible for antibiotic activation	*KatG*	*KatG*
Enzymes involved in antibiotic inactivation	*AAC(3)-II, AAC(3)-III, AAC(3)-IV, AAC(3)-VI, AAC(3)-VIII, AAC(3)-IX, AAC(3)-X, CatA1/CatA4 family, CatB family, CTX-M family, EreA family, Mph(A) family, NDM family, OXA-1 family, OXA-48 family, SHV family, TEM family*	*APH(3′')-I, OKP-A family, SHV family*
Gene clusters, cassettes, or operons associated with antibiotic resistance	*MarA, MarB, MarR*	*MarA, MarB, MarR*
Targets of antibiotics in susceptible species	*Alr, Ddl, dxr, EF-G, EF-Tu, folA, Dfr, folP, gyrA, gyrB, inhA, fabI, Iso-tRNA, kasA, MurA, rho, rpoB, rpoC, S10p, S12p*	*Alr, Ddl, Dxr, EF-G, EF-Tu, FolA, Dfr, FolP, GyrA, GyrB, InhA, FabI, Iso-tRNA, KasA, MurA, Rho, RpoB, RpoC, S10p, S12p*
Proteins protecting antibiotic targets	*Erm(B)*	*BcrC, QnrB family*
Efflux pumps that mediate antibiotic resistance	*BcrC*	*AcrAB-TolC, AcrAD-TolC, AcrEF-TolC, AcrZ, EmrAB-TolC, EmrD, MacA, MacB, MdfA/Cmr, MdtABC-TolC, MdtL, MdtM, SugE, Tet(D), TolC/OpmH*
Genes contributing to resistance through protein absence	*AcrAB-TolC, AcrAD-TolC, AcrEF-TolC, AcrZ, CmlA family, EmrAB-TolC, EmrD, MacA, MacB, MdfA/Cmr, MdtABC-TolC, MdtL, MdtM, QacE, SugE, TolC/OpmH*	*GidB*
Proteins that modify cell wall charge to confer resistance	*GidB*	*GdpD, PgsA*
Proteins regulating membrane permeability to antibiotics	*GdpD, PgsA*	*OccD6/OprQ, OprB*
Regulators influencing the expression of resistance genes	*OccD6/OprQ, OprB*	*AcrAB-TolC, EmrAB-TolC, H—NS, OxyR*

### 3.5 Comparative analysis and distribution of virulence genes in local *K. pneumoniae* isolates

Comparative genomics analysis identified multiple virulence associated determinants among the examined isolates, particularly isolate 5 (*K. pneumoniae* ST14). These included genes related to adhesion, iron acquisition system, capsule–associated regulator (*rmpA* and *rcsAb*), and secretion system components as shown in the Table 5. Genes encoding Type 1 and Type 3 fimbriae were detected in the analyzed isolates. In addition, Siderophore-associated genes including (aerobactin, salmochelin, and yersinibactin) were identified among examined isolates. Capsule-associated regulatory genes were also detected in the dataset. The distribution of virulence-associated genes identified in the two isolates is summarized in Table 5. Isolate 6 (*K. quasipneumoniae* ST7604) showed a different set of virulence-associated genes compared with isolate 5, including the absence of several siderophore-associated genes and adherence-related genes clusters.

**Table 5:** Virulence genes distribution among isolate 5 (*K. pneumoniae* ST14) and isolate 6 (*K. quasipneumoniae* ST7604) It illustrates the virulence factors, virulence classes and presence or absence (+/–) of the corresponding genes*.*

Gene	Virulence factor	Virulence class	isolate 5 (*K. pneumoniae* ST14)	isolate 6 (*K. quasipneumoniae* ST7604)
*mrkA, mrkB, mrkC, mrkD, mrkF, mrkH mrkI, mrkJ*	Type 3 fimbriae	Adherence	+	+
*fimA, fimB, fimC, fimD, fimE, fimF, fimG, fimH, fimI, fimK*	Type 1 fimbriae	Adherence	+	-
*PilW*	Type IV pili (Yersinia-like)	Adherence	+	-
*Capsule genes*	Capsule	Antiphagocytosis	+	+
*acrA, acrB*	AcrAB	Efflux pump	+	-
*iucA, iucB, iucC, iucD, iutA*	Aerobactin	Iron uptake	+	+
*iroB, iroC, iroD, iroE, iroN*	Salmochelin	Iron uptake	+	-
*fyuA, irp1, irp2, ybtA, ybtE, ybtP, ybtQ, ybtS, ybtT, ybtU, ybtX*	Yersiniabactin	Iron uptake	+	-
*allA, allB, allC, allD, allR, allS*	Allantoin utilization	Nutritional factor	+	+
*rcsA, rcsB*	RcsAB	Regulation	+	-
*rmpA*	RmpA	Capsule regulation	+	+
*T6SS-I genes*	T6SS-I	Secretion system	+	-
*T6SS-II genes*	T6SS-II		+	+
*T6SS-III genes*	T6SS-III		+	+
*LPS rfb locus*	LPS	Serum resistance	-	-
*clbA, clbB, clbC*	Colibactin	Toxin	-	-

### 3.6 Phylogenomic tree local *K. pneumoniae* isolates

**Fig. 2:**
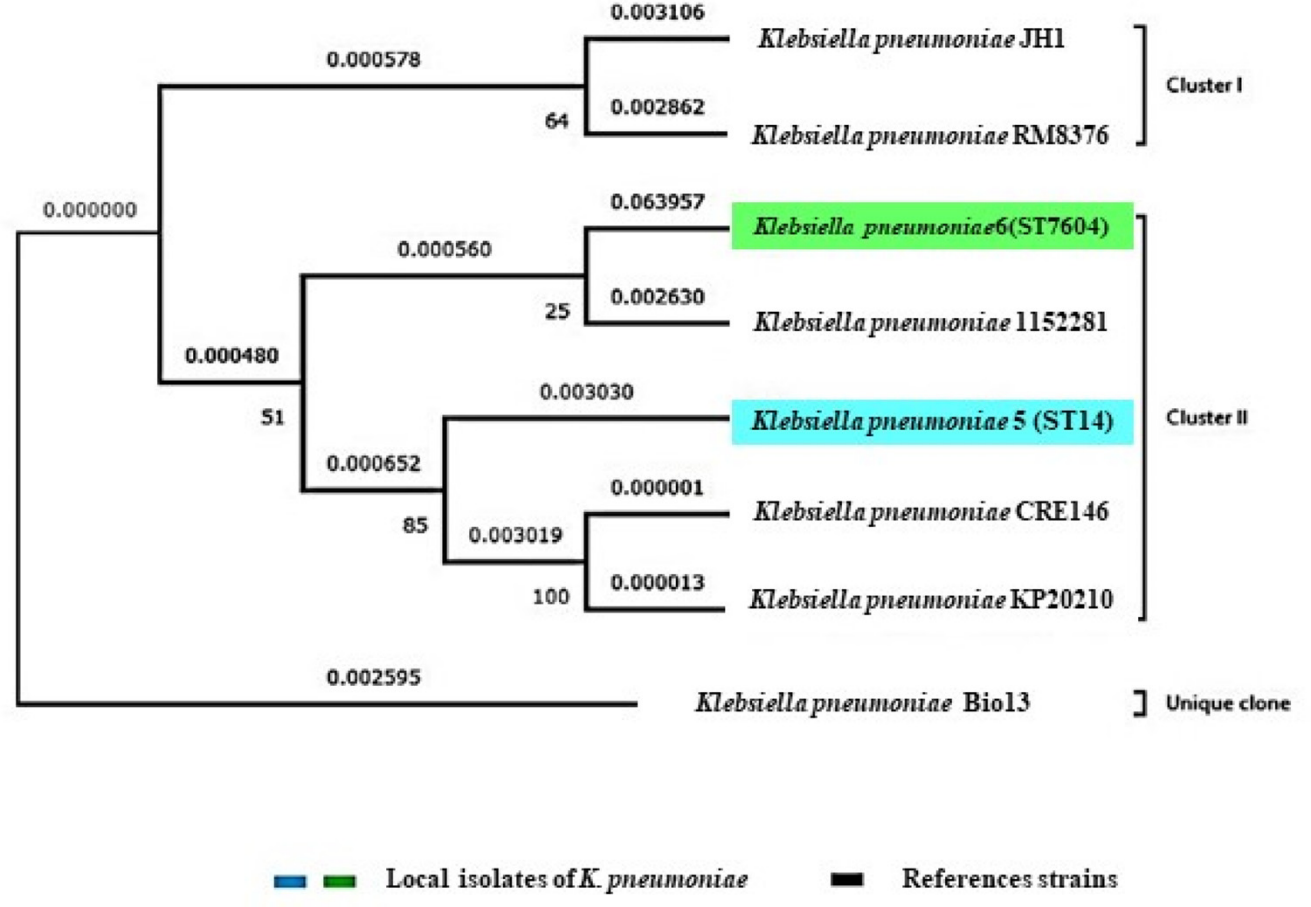
Phylogenetic tree of local isolate 5 (*K. pneumoniae* ST14) and isolate 6 (*K. quasipneumoniae* ST7604), (labelled with blue and green colors) compared with international reference strains from NCBI. The tree exhibited two main clusters: (Cluster I) refers to (reference strains JH1 and RM8376) and Cluster II including (examined local isolates), along with strains (1162,281, CRE146, and KP2010). The strain of *K. pneumoniae* Bio13 formed a distinct lineage (Unique clone). Genetic distances display evolutionary divergence among isolates.

Fig. 2 illustrates the phylogenomic tree constructed to compare the genomic relatedness of local isolates 5 and 6 (*K. pneumoniae* ST14 and *S. quasipneumoniae* ST7604) with reference strains available in the NCBI database. The phylogenomic tree shows two major clusters: Cluster I includes reference strains *K. pneumoniae* JH1 and RM8376, whereas Cluster II includes the clinical isolate 5 (*K. pneumoniae* ST14) and isolate 6 (*S. quasipneumoniae* ST7604) together with *K. pneumoniae* strains 1162,281, CRE146, and KP2010.

Isolate 6 (*K. quasipneumoniae* ST7604) showed a genetic distance value (0.063957) relative to the analyzed strains, while isolate 5 (*K. pneumoniae* ST14) clustered with strains CRE146 and KP2010 with in cluster II. In addition *K. pneumoniae* Bio13 formed a separate branch outside the two major clusters with a genetic distance value of (0.002595). Bootstrap value associated with the nodes of the phylogenomic tree are shown in Fig. 2.

## 4 Materials and Methods

### 4.1 Isolation and identification of bacterial isolates

A total of 115 urine samples were collected from patients with clinically suspected urinary tract infections in some Baghdad hospitals, which included Baghdad medical city, surgical specialties hospital and private nursing home hospital between October 2024 and January 2025. A total of non-duplicate 30 of the *K. pneumoniae* isolates were recovered from these urine samples based on standard microbiological culture, biochemical characterization, and confirmation using the VITEK-2 Compact system. The Bacterial isolates were subsequently cultured on both MacConkey agar (Himedia-India) and then on Eosin Methylene Blue agar (Himedia-India), at 37 °C for 24 h under aerobic conditions. Colony morphology—including color, shape, edges, and texture—was subsequently examined. All isolates were subjected to antimicrobial susceptibility testing, and multidrug resistance was defined according to CLSI criteria. Isolates No 5 (carbapenem-resistant) and No 6 (carbapenem-susceptible) were selected for whole-genome sequencing to represent contrasting resistance phenotypes

### 4.2 Antimicrobial susceptibility testing

The antimicrobial susceptibility testing was carried out against 13 different antibiotics via the disk diffusion method on 30 *K. pneumoniae* isolates. The interpretation of the results followed the Clinical and Laboratory Standards Institute (CLSI, 2024) guidelines [11]. A bacterial suspension was inoculated onto Mueller‒Hinton Agar plates (Oxoid, England), antibiotic disks were placed on the surface. Zones of growth inhibition were measured in millimeters after an overnight incubation. The antibiotics used were gentamycin (10 µg), imipenem (10 µg), meropenem (10 µg), cefotaxime (30 µg), ciprofloxacin (10 µg), aztreonam (30 µg), ceftriaxone (30 µg), ceftazidime (30 µg), amikacin (10 µg), levofloxacin (5 µg), nitrofurantoin (100 µg), ampicillin (10 µg) and trimethoprim/sulfamethoxazole (25 µg), all antibiotics were purchased from (Bioanalyse, Turkey). The Vitek-2 compact system (Bio Merieux, France) involves the preparation of the inoculum, a (0.45 %) of saline solution in which the *K. pneumoniae* adjusted to the standard turbidity (0.5 McFarland) and used for the determination of the MIC toward different antibiotics (AST).

### 4.3 Extraction of bacterial genomic DNA and genome sequencing

Genomic DNA from the local *K. pneumoniae* isolates was extracted using a commercial extraction kit (Roche Applied Science, Mannheim, Germany), following the manufacturer’s instructions. DNA quality and integrity were evaluated by electrophoresis on a 1 % agarose gel, and DNA concentration was quantified using fluorometry (Qubit, Life Technologies, Carlsbad, USA).

DNA Sequencing libraries were prepared using the illumina TruSeq Nano DNA library preparation kit (Illumina Inc., San Diego, CA, USA) following the manufacture’s protocol. DAN quantity using and purity were evaluated using a NanoDrop 2000 spectrophotometer (Thermo Fisher Scientific, Wilmington, DE, US). Whole-genome sequencing was performed using Illumina MISeq sequencing platform (Illumina Inc., San Diego, CA, USA) with the MiSeq Reagent Kit v2, producing paired-end reads 2 × 250 bp. The sequencing reads were subjected to quality control and adapter trimming prior to downstream genome assembly and bioinformatics analysis [12]. Raw sequencing reads generated from Illumina MISeq sequencing platform were initially assessed for quality using FastQC (v0.11.9). Adaptor sequencing and low-quality bases were trimmed using Trimmomatic (v0.39). High quality reads subsequently assembled de novo using SPAdes genome assemble (v3.15.5) with default parameters. The quality and completeness of the assembled genomes were evaluated using CheckM (v1.2.2) to assess genome completeness and potential contamination. Genomic annotation was performed using Prokka (v1.14.6) to identify coding sequencing and functional genomic features. Multilocus sequences typing (MLST) analysis was conducted using the MLST tool available at the Center for the Genomic Epidemiology to determine sequence types of the local isolates.

High quality reads were de novo assembled using unicycler, which is optimized for bacterial genome assembly strategies to improve contiguity and accuracy. Genomic annotation was performed using the NCBI prokaryotic genome annotation pipeline (PGAP) with default parameters to ensure standardized gene prediction and functional annotation. In parallel, assembled genomes were uploaded to PATRIC server for functional categorization and identification of antimicrobial resistance and virulence- associated genes [13].

### 4.4 Screening for antimicrobial resistance genes (AMR) and virulence factors (VF) among examined isolates

Multilocus sequence typing (MLST) was achieved in silico using MLST 2.0. *Klebsiella* pathogenicity islands (KPIs) were determined using KPIFinder 1.0. The presence of AMR genes was determined using ResFinder 4.1 (https://cge.food.dtu.dk/services/ResFinder/), and the comprehensive antibiotic resistance database (CARD) v1.1.4 [14]. The prediction of virulence (VR) genes was investigated by the VF of Pathogenic Bacteria (VFDB) platform [15]. The circular genomic map was constructed with BLAST and using standard parameters.

### 4.5 Phylogenomic tree

Phylogenomic tree which was constructed to evaluate the evolutionary relationships between local isolates (*K. pneumoniae* ST14 and *S. quasipneumoniae* ST7604) in comparison with reference strains deposited in NCBI. PATRIC global protein families (PGFams) from these genomes were selected to determine the phylogenetic placement of the analyzed genome. Protein sequences from these families were aligned using MUSCLE [16], and the corresponding nucleotide sequences were mapped to the protein alignment. The resulting amino acid and nucleotide alignments were concatenated into a single data matrix, which was analyzed using RAxML [17]. Fast bootstrapping [18] was employed to generate support values for the phylogenetic tree.

## Limitations

Not applicable

## Ethics Statement

Ethics approval this study was approved from University of Baghdad, College of Science for women (Approval Ref: CSW-REC 1013, Date: 11/12/2025). The study involved minimal-risk anonymized urine samples collected as part of routine clinical practice. Formal approval was issued after initiation of sample collection due administrative delays. The study had been conducted under the supervision and permission of the department, and no identified patient information was collected. All procedures were performed in accordance with ethical standard of the institutional committee and declaration of Helsinki.

## CRediT Author Statement

**Hijran M. Meshjel:** Sample collection, Methodology, Writing-review & editing, Writing-original draft; **Safaa A. A. S. Al-Qaysi:** Supervision, Project administration, Writing-review &editing.

## Data Availability

Mendeley DataRaw genome sequencing data of the Klebsiella pneumoniae clinical isolates (Original data). Mendeley DataRaw genome sequencing data of the Klebsiella pneumoniae clinical isolates (Original data).

## References

[1] Ferri. E. Ranucci M., Romagnoli P., Giaccone V. (2017). Antimicrobial resistance: a global emerging threat to public health systems. Crit. Rev. Food Sci. Nutr..

[2] Authority; European Food Safety (2015). European centre for disease prevention and control. EU summary report on antimicrobial resistance in zoonotic and indicator bacteria from humans, animals and food in 2013. EFSa J..

[3] Bassetti M., Righi E., Carnelutti A., Graziano E., Russo A. (2018). Multidrug-resistant *Klebsiella pneumoniae*: challenges for treatment, prevention and infection control. Expert Rev. Anti-Infect. Ther..

[4] Pendleton J.N., Gormanm S.P., Gilmore B.F. (2013). Clinical relevance of the ESKAPE pathogens. Expert Rev. Anti-Infect Ther..

[5] Bengoechea J.A., Sa Pessoa J. (2019). *Klebsiella pneumoniae* infection biology: living to counteract host defences. FEMS Microbiol. Rev..

[6] Li B., Zhao Y., Liu C., Chen Z., Zhou D. (2014). Molecular pathogenesis of Klebsiella pneumoniae. Future Microbiol..

[7] Chegini Z., Khoshbayan A., Vesal S., Moradabadi A., Hashemi A., Shariati A. (2021). Bacteriophage therapy for inhibition of multidrug-resistant uropathogenic bacteria: a narrative review. Ann. Clin. Microbiol. Antimicrob..

[8] Elbehiry A., Marzouk E., Abalkhail A. (2026). Molecular insights into carbapenem resistance in *Klebsiella pneumoniae*: from mobile genetic elements to Precision diagnostics and infection control. Int. J. Mol. Sci..

[9] Lam L., Holt M.M.C. (2020). Population genomics of *Klebsiella Pneumoniae*. Nat. Rev. Microbiol..

[10] Navon-Venezia S., Kondratyeva K., Carattoli A. (2017). *Klebsiella Pneumoniae*: a major worldwide source and shuttle for antibiotic resistance. FEMS Microbiol. Rev..

[11] CLSI (2023).

[12] Grant J.R., Enns E., Marinier E., Mandal A., Herman E.K., Chen C.Y., Graham M., Van Domselaar G., Stothard P. (2023). Proksee: in-depth characterization and visualization of bacterial genomes. Nucleic. Acids. Res..

[13] Wick R.R., Judd L.M., Gorrie C.L., Holt K.E. (2017). Unicycler: resolving bacterial genome assemblies from short and long sequencing reads. PLoS Comput. Biol..

[14] McArthur A.G., Waglechner N., Nizam F., Yan A., Azad M.A., Baylay A...J.., Bhullar K., Canova M.J., De Pascale G., Ejim L., Kalan L., King A.M., Koteva K., Morar M., Mulvey M.R., O’Brien J.S., Pawlowski A.C., Piddock L.J.V., Spanogiannopoulos P., Sutherland A.D., Tang I., Taylor P.L., Thaker M., Wang W., Yan M., Yu T., Wright G.D. (2013). The comprehensive antibiotic resistance database, antimicrob. Agents Chemother..

[15] Liu B., Zheng D., Zhou S., Chen L., Yang J. (2022). VFDB 2022: a general classification scheme for bacterial virulence factors. Nucleic. Acids. Res..

[16] Edgar Robert R.C. (2004). MUSCLE: multiple sequence alignment with high accuracy and high throughput. Nucleic. Acids. Res..

[17] Stamatakis A. (2014). RAxML version 8: a tool for phylogenetic analysis and post-analysis of large phylogenies. Bioinformatics..

[18] Stamatakis A., Hoover P., Rougemont J. (2008). A rapid bootstrap algorithm for the RAxML web servers. Syst. Biol..

